# Genome-wide identification and characterization of *SRLK* gene family reveal their roles in self-incompatibility of *Erigeron breviscapus*

**DOI:** 10.1186/s12864-023-09485-0

**Published:** 2023-07-17

**Authors:** Chenggang Xiang, Hongzheng Tao, Tiantao Wang, Hengling Meng, Dejun Guan, He Li, Xiang Wei, Wei Zhang

**Affiliations:** 1 Honghe University, Mengzi, 661100 Yunnan China; 2 Yunnan Zesheng Biotechnology Co., Ltd. Luxi, Qujing, 652400 Yunnan China; 3 Key Laboratory of Ethnomedicine, Ministry of Education, Minzu University of China), Beijing, 100081 China

**Keywords:** *SRLK* gene family, Self-incompatibility, *E. breviscapus*, Gene expression

## Abstract

Self-incompatibility (SI) is a reproductive protection mechanism that plants acquired during evolution to prevent self-recession. As the female determinant of SI specificity, *SRK* has been shown to be the only recognized gene on the stigma and plays important roles in SI response. Asteraceae is the largest family of dicotyledonous plants, many of which exhibit self-incompatibility. However, systematic studies on *SRK* gene family in Asteraceae are still limited due to lack of high-quality genomic data. In this study, we performed the first systematic genome-wide identification of *S*-locus receptor like kinases (*SRLKs*) in the self-incompatible Asteraceae species, *Erigeron breviscapus*, which is also a widely used perennial medicinal plant endemic to China.52 *SRLK* genes were identified in the *E. breviscapus* genome. Structural analysis revealed that the EbSRLK proteins in *E. breviscapus* are conserved. SRLK proteins from *E. breviscapus* and other SI plants are clustered into 7 clades, and the majority of the EbSRLK proteins are distributed in Clade I. Chromosomal and duplication analyses indicate that 65% of the *EbSRLK* genes belong to tandem repeats and could be divided into six tandem gene clusters. Gene expression patterns obtained in *E. breviscapus* multiple-tissue RNA-Seq data revealed differential temporal and spatial features of *EbSRLK* genes. Among these, two *EbSRLK* genes having high expression levels in tongue flowers were cloned. Subcellular localization assay demonstrated that both of their fused proteins are localized on the plasma membrane. All these results indicated that *EbSRLK* genes possibly involved in SI response in *E. breviscapus.* This comprehensive genome-wide study of the *SRLK* gene family in *E. breviscapus* provides valuable information for understanding the mechanism of SSI in Asteraceae.

**Supplementary Information:**

The online version contains supplementary material available at 10.1186/s12864-023-09485-0.

## Introduction

Self-incompatibility (SI) is a reproductive mechanism in angiosperm plants to prevent inbreeding by inhibiting self-pollination [1, 2]. SI has been widely observed and documented in plant families such as Brassicaceae [3], Solanaceae [4], Rosaceae [5], Plantaginaceae [6], Scrophulariaceae [7], Poaceae [8], and Rutaceae [9]. Based on the genetic patterns of pollen incompatibility phenotypes, SI is classified as gametophytic self-incompatibility (GSI) and sporophytic self-incompatibility (SSI) [1]. In SSI, the molecular mechanism of SI is well elucidated in detail in the *Brassica* genus of the Brassicaceae family [10, 11]. SSI represented by Brassica is controlled by a multi-allelic S locus, which has multiple genes. Among them, there are three key multiple alleles in controlling SI, namely the S-locus receptor kinases (SRKs), the S-locus glycoprotein (SLG) and the S-locus cysteine-rich protein (SCR).

SRKs belong to the family of plant receptor protein kinases and are generally localized on the cell membrane with functions of signal perception and recognition. The SRK protein is specifically expressed in the papillary cells of the stigma. The SRK family comprises of 10 subfamilies, which are reported to play important roles in SI of different species [12]. Typical SRKs have three domains: an N terminal extracellular domain, a transmembrane domain, and a C terminal cytoplasmic kinase domain exhibiting serine/threonine protein kinase activity. The extracellular domain of SRKs shares extensive sequence identity with SLG, thus also called SLG-like ectodomain (eSRK) [13]. eSRK consists of two lectin domains with 12 conserved cysteine residues, six in the EGF-like domain, and six in the PAN domain [14, 15]. SRK is a highly polymorphic protein among the haplotypes, with three hypervariable (HVI–III) regions, which are important for perception specificity in SI response [15, 16].

Functional complementation confirms the participation of SLGs and SRKs in SI response [17]. By transforming self-incompatible plants of *Brassica napa* with an SRK28 and an SLG28 transgene separately, Takasaki et al. (2000) confirmed that expression of SRK28 alone, but not SLG28 alone, conferred the ability to reject self (S28)-pollen on the transgenic plants. They also showed that the ability of SRK28 to reject S28 pollen was enhanced by SLG28, suggesting that SRK alone determines the S haplotype specificity of the stigma, and that SLG promotes a full manifestation of the self-incompatibility response [17]. The role of SRK as the female determinant of SSI is further supported by the functional verification of SRK in *Brassica oleracea* L. [2]. In addition to the above mentioned components, other proteins such as Arm repeat-containing protein 1 (ARC1), thioredoxin H-Like proteins (THL1/THL2), Aquaporin, Exo70A1 and M-Locus protein kinase (MLPK) are also involved in SSI [17–19].

Genetic studies of SI in Asteraceae started with species *Crepis foetida* L. [20], guayule [21], and annual chrysanthemum [22] in the 1950s and 1960s. A literature survey on the breeding system of 571 species in the Asteraceae family showed that most (63%) of the Asteraceae plants are self-incompatible [23]. *Senecio squalidus* L. is a model species for studying SI in Asteraceae. Studies on *S. squalidus* indicate that SI in Asteraceae belongs to the SSI system and is controlled by an S locus [24–28]. Three *SRK*-like (*SRLKs*) cDNAs were amplified from the stigma of the heterozygous S (1) and S (2) S alleles of *S. squalidus*. However, the expression patterns of these *SRLKs* are different from the patterns of typical SSI in Cruciferae, suggesting that the *S. squalidus* SRLKs unlikely directly participate in SI or pollen-stigma interaction [29]. The results of cellular and molecular studies on *S. squalidus* revealed that although they share the same SSI genetic system, the molecular mechanisms of Cruciferae and Asteraceae are different [29]. Despite some explorations have been carried out in *S. squalidus*, the SI mechanism in Asteraceae is still elusive.

*Erigeron breviscapus* (Vaniot) Hand.-Mazz., which belongs to the genus *Erigeron* of the Asteraceae family, is a genuine medicinal plant in southwest China. In a previous study, we performed a comparative transcriptomic analysis in the capitulum of *E. breviscapus* plants under different pollination treatments. In the three cDNA libraries (non-pollinated, self-pollinated and cross-pollinated of capitulum of *E. breviscapus*), approximately 230 differentially expressed genes that may be related to the SI response were identified. For example, *SRLK* and its downstream signaling factor *MLPK* are up-regulated in self-pollinated flowers, but down-regulated in cross-pollinated flowers [30]. Based on the transcriptomic data, using RACE techniques, we cloned the full-length *EbSRLK1* gene in *E. breviscapus*. In addition, quantitative real-time PCR (qRT-PCR) analysis showed that the *EbSRLK1* gene is lowly expressed in roots, stems, and leaves, but highly expressed in flowers, especially in the flowers on the day before opening. This study provides preliminary information for investigating the SI mechanism in *E. breviscapus* and Asteraceae [30].

In this study, in order to explore the roles of SRLK in controlling SI in Asteraceae, we systematically identified the *SRLK* gene family in the *E. breviscapus* genome and selected the *SRLK* genes from the typical GSI plant *Pyrus spp. communis* (Rosaceae family) and from the typical SSI plant *B.oleracea* (Brassicaceae family) for phylogenetic comparison. The conserved domain analysis of EbSRLKs was performed and the expression patterns of *EbSRLKs* in different tissues/organs of *E. breviscapus* and at different flowering stages or pollination conditions were compared. This study provides valuable information for future studies to understand the SI mechanism in *E. breviscapus* and more broadly in Asteraceae.

## Materials and methods

### Plant materials and growth conditions

The *Erigeron breviscapus* seedlings used in this study were provided by Professor Shenchao Yang at Yunnan Agricultural University. Since 2020, seedlings were cultivated in the greenhouse of Honghe University under a photoperiod of 14 h light (25℃) and 10 h dark (16℃), with 200 ~ 600 umol/m^2^/s photosynthetic photon flux density and 65 ~ 80% humidity for 2 months, and then transferred to a photoperiod of 10 h light (25℃) and 14 h dark (16℃) to induce flowering. Based on the flower morphology, the entire flowering process was divided into 6 developmental stages (Fs1 to Fs6).

### Identification of the *SRK *family members in *Erigeron breviscapus*

Because the *Arabidopsis* SRKs are well documented, they were used as reference sequences. All the sequences of *A. thaliana* SRK proteins (AtSRKs) were downloaded from The *Arabidopsis thaliana* (L.) Heynh. Information Resource (TAIR). The whole genome sequence of *E. breviscapus* was downloaded from the GigaScience Database (http://gigadb.org/). For identification purposes, AtSRKs were used as query sequences, and BLASTP was performed (using the NCBI-BLAST-2.7.1 + program) against the *E. breviscapus* genome. The e-value was set to 1E^−5^. For the correct identification, more than 60% of similar sequences were included. These identified candidates were further filtered through domain analysis to ensure that the selected sequences are non-redundant sequences to determine the true SRLK family members. For the domain analysis, the standard HMM profiles of the modular S-domain, which includes the B_lectin (PF01453), PAN (PF08276) and SLG (PF00954), were downloaded from the Pfam database (http://pfam.xfam.org/) and then searched against the local protein database of *E. breviscapus*. All candidate members should contain one of the Pfam domain model. Lastly, one or more transmembrane helices (TMHs) should be present to ultimately identify SRLK family members. The candidate SRLKs were confirmed by the SMART web server (http://smart.emblheidelberg.del) and the CD-search (https://www.ncbi.nlm.nih.gov/Structure/cdd/wrpsb.cgi).

### Multiple sequence alignment and construction of phylogenetic tree

To evaluate the phylogenetic relationship of *EbSRLK* with *SRK* genes from other self-incompatible species, we chose the typical sporophytic self-incompatibility species *B. oleracea* and the typical gametophytic self-incompatibility species *Pyrus spp. communis*. The annotated protein sequences in the sequenced genomes of *B. oleracea* and *Pyrus spp. communis* were downloaded from the Ensembl Genomes Database (http://plants.ensembl.org/index.html) and the Rosaceae Genome Database (https://www.rosaceae.org), respectively. The same methods that were used for identifying EbSRLKs were used to identify the SRLKs in *B. oleracea* (BoSRLK) and *P. spp communis* (PsSRLK). The full-length SRLK protein sequences of EbSRLK*,* BoSRLK and PsSRLK were aligned using the ClustalX 2.0 program with the default parameters [31]. The phylogenetic tree was constructed using the Maximum-Likelihood (ML) method and JTT substitution model of the MEGA7.0 software with 1000 bootstrap replicates.

### Characterization of EbSRLK genes and proteins

The CDS and protein sequences of EbSRLKs were extracted using the TBtools [32]. To illustrate the structures of *EbSRLK* genes, the coding sequence of each *SRLK* gene was aligned with its genomic sequence using the Gene Structure Display Server (GSDS) program (http://gsds.gao-lab.org/). The protein isoelectric point (pI) and molecular weight (MW) of *EbSRLK* proteins were predicted using the ExPASy proteomics server database (https://www.expasy.org/) and the protein sequence identities of the SRLKs were analyzed using BioEdit [33]. Motifs were identified using the MEME program (http://meme-suite.org/). The parameters were set as zero or one occurrence (of a contributing motif site) per sequence, and the numbers of motif were chosen as five motifs; the motif width was set to 6 to 50. Putative transmembrane helices in proteins were predicted using TMHMM 2.0 web server (https://services.healthtech.dtu.dk/service.php?TMHMM-2.0).

### Chromosomal distribution and duplication events of *EbSRLK* genes

The chromosomal location of all putative *EbSRLK* genes were analyzed using a local BLAST program [34] and visualized using the OmicCircos R package [35]. The gene density and the syntenic relationship in the *E. breviscapus* genome were analyzed using the RIdeogram R package [36] and MCScanX, respectively [37]. Gene duplication events were analyzed manually, and tandem duplications were characterized as multiple members in one family occurring within the same intergenic region or in neighboring intergenic regions [38]. Major criteria used for analyzing potential gene tandem duplication included the length of aligned sequence coverage > 75% of the longer gene and the similarity of aligned region > 75% [39]. *Ka* and *Ks* value were calculated using KaKs Calculator [40].

### Expression analysis of *EbSRLKs* under different pollination treatments and in different tissues

The expression profiles of *EbSRLKs* were obtained by analyzing RNA-Seq data downloaded from the *Erigeron breviscapus* genome database (http://gigadb.org/dataset/100290), including sequences data from multiple tissues. The RNA-Seq data for self-pollinated and cross-pollinated flowers were downloaded from NCBI (SRR1867750). The gene expression levels were estimated by the TopHat/Cufflinks pipeline described in the previous reports with FPKM (fragments per kilobase of exon per million fragments mapped) values. The heat maps for expression profiles in different tissues and at different flowering stages were generated with the OmicShare Tools (http://www.omicshare.com/tools/Home/Index/index.html).

For the expression analysis of *EbSRLK* genes by quantitative real-time PCR (qRT-PCR), total RNA was isolated from indicated *E. breviscapus* tissues using TRIzol reagent (Invitrogen, USA) according to the manufacturer’s instructions. First-strand cDNA synthesis was performed using a PrimeScript™ RT Reagent Kit with gDNA Eraser (TaKaRa, Japan). qRT-PCR was carried out using an ABI 7500 Real-time PCR System (Bio-Rad, USA) with SYBR® Premix Ex Taq™ (TaKaRa, Japan). The Beacon Designer 8.14 program was used to design the primers (Premier Biosoft International, Palo Alto, CA). The *E. breviscapus ACTIN* gene was used as the reference control. Primers used in qRT-PCR were listed in Supplementary Table S6. Changes in gene expression were calculated using the 2^ΔΔ*Ct*^ method [41]. For each qRT-PCR experiment, three biological replicates of each tissue sampled were used. For each biological replicate, three technical replicates were used.

### Subcellular localization prediction and confirmation

The subcellular localization of the 52 EbSRLK proteins were predicted using the online predictor server CELLO v2.5 (http://cello.life.nctu.edu.tw/). The full length CDS sequences (without stop codon) of *EbSRLK23* and *EbSRLK43*, which exhibit high expression levels in most tissues and flowering stages, were amplified. The PCR products were ligated to the pEGOEP35S-eGFP plasmid by T4 DNA ligase (TaKaRa, Japan) to generate fusion expression vectors. The *Arabidopsis thaliana* (L.) Heynh protoplasts were prepared following the method described by Abel and Theologis [42] and the *35S::EbSRLKs::eGFP* and *35S::eGFP* plasmids were introduced into protoplasts using the PEG-mediated method [43]. To further confirm the subcellular localization results generated in protoplasts, transient transformation of epidermal cells of tobacco leaves was employed [44]. The *Agrobacterium tumefaciens* (strain GV3101) suspension that contains one of the above recombinant vectors was infiltrated into 5-week-old tobacco leaves. Seventy-two hours later, tobacco leaves were removed from the plant and the GFP fluorescence was visualized using the LSM800 confocal microscopy imaging system (Zeiss Co., Oberkochen, Germany).

## Results

### Identification of SRLKs and phylogenetic analysis

To evaluate the phylogenetic relationship of *EbSRLKs* with *SRLK* genes in other self-incompatible species, we chose the typical sporophytic self-incompatibility species *B.oleracea* and the typical gametophytic self-incompatibility species *Pyrus communis* for SRLK identification. The S domain (SD), which includes three subdomains with a configuration of B_lectin-SLG-PAN, is the key feature of SRLKs and was used for SRLK identification. Using standard HMM profiles, 50 sequences from *B.oleracea*, 63 sequences from *Pyrus communis* and 53 sequences from *E. breviscapus* with the typical SD domain architecture were identified (Supplementary Table S1). Proteins with stand-alone SLG and PAN domains were rare, whereas the B_lectin domain was more abundant in the genomes of the three species (Supplementary Fig. 1a). The sequences identified based on SD were then uploaded to the Pfam database to search for kinase domains (KD). After excluding 22 (*B.oleracea*), 15 (*Pyrus communis*) and 1 (*Erigeron breviscapus*) sequences that had no kinase domains, the final identified SRLKs in the three species were 28 BoSRLKs, 48 PsSRLKs and 52 EbSRLKs (Supplementary Fig. 1a). The domain architectures were classified into 26 types based on combinations of B_lectin, SLG, PAN and KD domains. Among these types, B_lectin-SLG-PAN-PK_tyr-ser-Thr was the dominant type of architecture in the three species (Supplementary Fig. 1b).

**Fig. 1:**
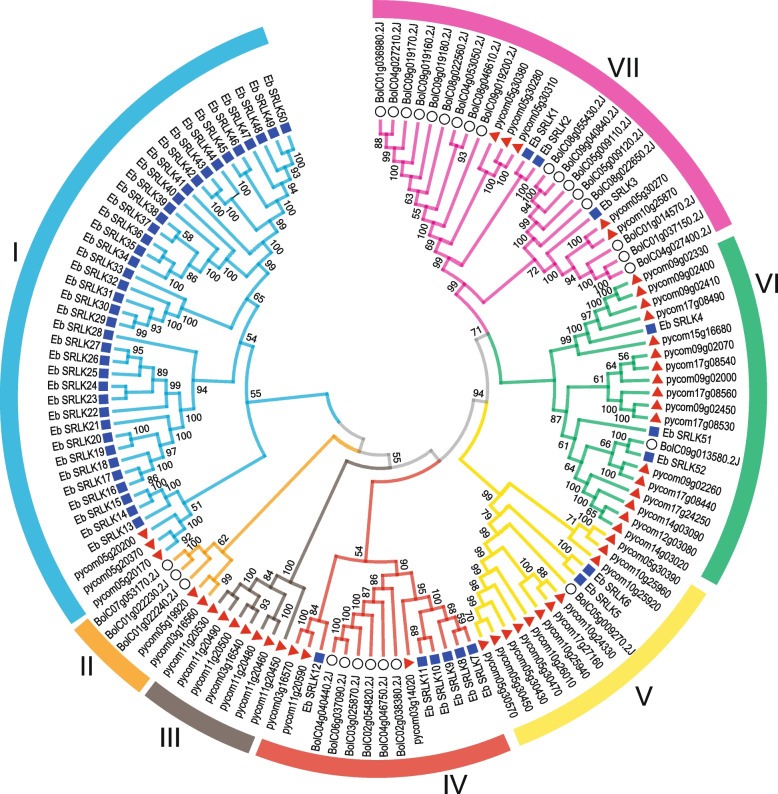
Phylogenetic relationship of putative *SRLK* genes from *Pyrus communis* (Pycom,  ), *Erigeron breviscapus* (Eb,  ), and *B.oleracea* (Bol,  ). The unrooted phylogenetic tree was constructed using the MEGA 7 software through the neighbor-joining (NJ) method with 1000 bootstrap replicates. The bootstrap values are shown near the nodes, and only those values greater than 50 are displayed. The seven groups are indicated with camber lines

In order to understand the evolutionary relationships between EbSRLKs and SRLKs from other two self-incompatibility species, the amino acid sequences of 28 putative BoSRLKs, 48 putative PsSRLKs and 52 putative EbSRLKs were aligned to construct an unrooted neighbor-joining phylogenetic tree. The results indicated that the SRLKs from the three species were classified into seven clades (Clade I to VII) (Fig. 1). SRLKs from different species were extremely unevenly distributed in different clades. For example, Clade I was the largest group with 41 members, 38 of them being EbSRLKs. Clade VII was formed predominantly by 17 BoSRLKs. Clade V and VI were formed predominantly by 11 and 17 PsSRLKs, respectively. Clade III had only 7 PsSRLKs, no other SRLKs from *E. breviscapus* or *B. oleracea*. The remaining 14 EbSRLKs were scattered in Clade IV, V, VI and VII. Similarly, 3, 6, 1, and 1 BoSRLKs were distributed in Clade II, IV, V and VI, respectively; and 3, 2, 3, and 5 PsSRLKs were scattered in Clade I, II, IV and VII, respectively. The results revealed considerable interspecific conservation and intraspecific diversification in different *SRLK* gene families.

### *EbSRLK* gene features, structures and conserved motifs analysis of EbSRLK proteins

**Fig. 2:**
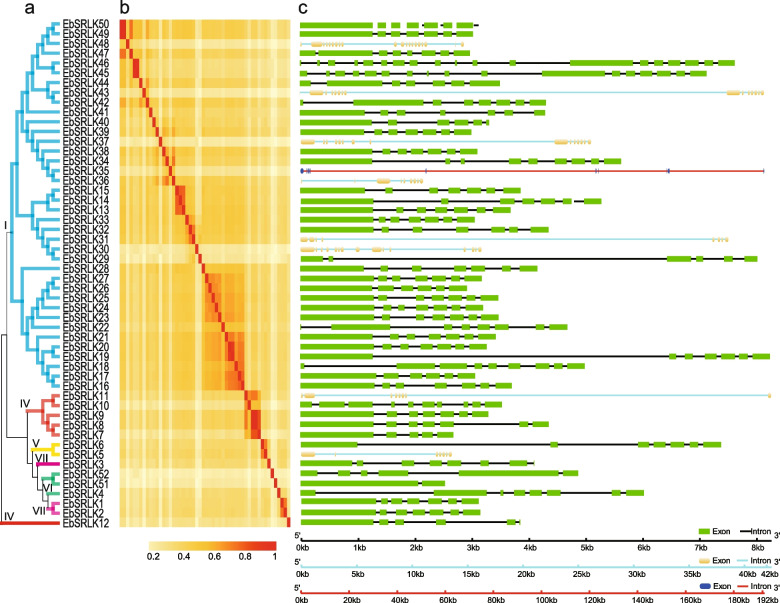
Phylogenetic relationships, protein sequence identities and gene structure of putative *SRLKs* in *Erigeron breviscapus*. **a** Neighbor-joining (NJ) phylogenetic unrooted tree for *SRLKs* in *Erigeron breviscapus*. Different colors represent the original grouping relationships between *SRLKs* from the three species in Fig. 1. **b** Protein sequence identities of SRLKs in *Erigeron breviscapus*. Heat map represents the protein sequence identities of SRLKs analyzed by ClustalW. The colored bar indicates 20–100% protein sequence identity. **c** Exon–intron structures of 52 *SRLKs* identified in *Erigeron breviscapus*. Due to the large difference in length between genes, three different scales are used to show the length of genes. Exons are presented by green, yellow and dark blue boxes in the three scales, and introns are represented by black, light blue and red lines in the three scales

Phylogenetic analysis was carried out using the amino acid sequences of the 52 EbSRLK proteins. The relationship among the 52 EbSRLKs proteins in the new phylogenetic tree (Fig. 2a) was basically similar to the results in the phylogenetic tree constructed in multiple species (Fig. 1). Levels of conservation among EbSRLKs proteins were analyzed via a heat map analysis of amino acid similarity. The results showed that amino acid similarities were consistent with the results of phylogenetic analysis (Fig. 2b). Using the coding sequence (CDS) information of 52 *EbSRLKs*, the gene structures were analyzed (Fig. 2c). The results indicated that the exon numbers of the *EbSRLK* genes ranged from 2 (*EbSRLK51*) to 20 (*EbSRLK48*) (Supplementary Table S2), with most *EbSRLK* genes containing 6–9 exons but a few genes containing more than 15 exons, such as *EbSRLK35*, *EbSRLK45*, *EbSRLK46* and *EbSRLK48*. The CDSs of the 52 *EbSRLK* genes ranged from 1,392 bp (*EbSRLK29*) to 5,052 bp (*EbSRLK37*), with a mean length of 2,644 bp. The genomic regions of most *EbSRLK* genes (43 genes) were less than 8.5 kb, while eight of the *EbSRLK* genes had genomic regions between 20 to 43 kb Exceptionally, *EbSRLK37* had a genomic region of 192 kb (Fig. 2c). Combined with the phylogenetic tree and protein similarity data, the results of gene structures indicated that the exon/intron structures were not highly conserved in each clade. The theoretical pI of the 52 EbSRLKs proteins ranged from 5.18 to 8.69. Subcellular localization prediction showed that 25, 11, 6, 5 and 4 of 52 *EbSRLK* proteins were predicted to localize on the plasma membrane, chloroplast, nucleus, vacuolar membrane and extracellular matrix, respectively (Supplementary Table S2).

Based on studies from Brassicaceae, SRKs have an extracellular S domain, which is responsible for ligand binding, and an intracellular kinase domain, which is responsible for transducing signals into cellular responses by phosphorylating the Arm Repeat Containing (*ARC*) proteins [15]. The S domain and the kinase domain is separated by one or more transmembrane domains (TMs). Therefore, the SRLKs in *Erigeron breviscapus* should contain a TM. To confirm the presence of TMs in 52 EbSRLKs, the protein sequences were submitted to the TMHMM Server for TMs prediction. The results showed that 46 of the 52 EbSRLKs contained at least 1 TMs, 6 of the 52 EbSRLKs (EbSRLK2, 4, 5, 35, 36, 40) had no TMs (Supplementary Table S2, Supplementary Figure S2).

The conserved motifs of the 52 EbSRLK proteins were analyzed using the online MEME software. A total of eight conserved motifs were identified (Supplementary Figure S3). Among these motifs, Motif 1 was found in the B_lectin domain region, and Motif 2 was found in the SLG domains, while Motif 4 and Motif 5 were found in the PAN domain. Basically, all 52 EbSRLK proteins contained at least 5 of the 8 conserved motifs, while a few proteins lacking some of the conserved motifs, for example, EbSRLK48 lacking Motif 2, EbSRLK41 lacking Motif 4, EbSRLK40 lacking Motif 8, EbSRLK29 and EbSRLK12 lacking Motif 6, Motif 7 and Motif 8.

### Chromosomal distribution and duplication events of *EbSRLK* genes

**Fig. 3:**
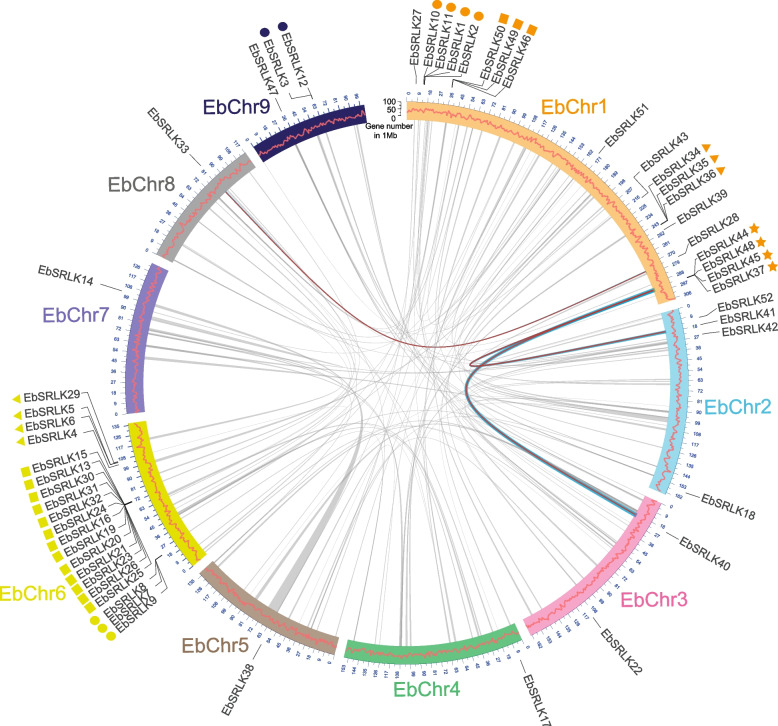
The distribution and inter-chromosomal correlation of the *SRLK* gene family in *Erigeron breviscapus*. The arc segments represent nine chromosomes of *Erigeron breviscapus*. The red curve in each arc segment indicates gene number in 1 Mb. The scale outside each chromosome represents the physical position and length of chromosome (Mb). The outermost symbols indicate tandem duplication genes, with same-shaped symbols representing one tandem duplication group. Triangles, squares and solid circles represent multiple tandem duplication gene group in same chromosome. The inner lines represent syntenic blocks detected in the genome, the blocks which harbored segmental duplication *SRLKs* are highlighted by blue line and the relationship between segmental duplication *SRLKs* are shown with red line

The 52 *EbSRLK* genes were unevenly distributed in the nine *Erigeron breviscapus* chromosomes, ranging from 1 to 20 genes per chromosome (Fig. 3, Supplementary Table S2). Chromosome 1 and 6 harbored the most number of *EbSRLK* genes (19 and 20 genes, respectively), whereas Chromosome 4, 5, 7, and 8 had only one *EbSRLK* gene on each chromosome.

Whole genome duplication (WGD), tandem and segmental duplication gave rise to gene families during biological evolution [45, 46]. To investigate the possible reason of *EbSRLK* gene expansion, we re-analyzed the *E. breviscapus* genome using MCScanX. The whole genome analysis did not yield significant evidence of whole-genome duplication but plenty of syntenic blocks were detected (Fig. 3, Supplementary Table S3). To confirm the effect of tandem duplication in *EbSRLK* gene family expansion, the Ka and Ks values of the gene pairs that are distributed in the same intergenic region or in the neighboring intergenic region were calculated. The analysis results indicated that 65% (34/52) of the *EbSRLK* genes belong to tandem repeats (Fig. 3, Supplementary Table S4). Based on the locations of these genes on chromosomes, the tandem repeat genes were divided into six tandem-gene clusters, with the largest cluster detected on Chromosome 6 with 13 *EbSRLK* genes (Fig. 3, Supplementary Table S2).

By integrating the chromosomal distribution of *EbSRLKs* and the syntenic block information, three pairs of segmental duplications were identified, including the *EbSRLK33* and *EbSRLK28* gene cluster, the *EbSRLK40* and *EbSRLK44* gene cluster, and the *EbSRLK41* and *EbSRLK44* gene cluster (Fig. 3, Supplementary Table S3).

### Expression profiles of *EbSRLK* genes

**Fig. 4:**
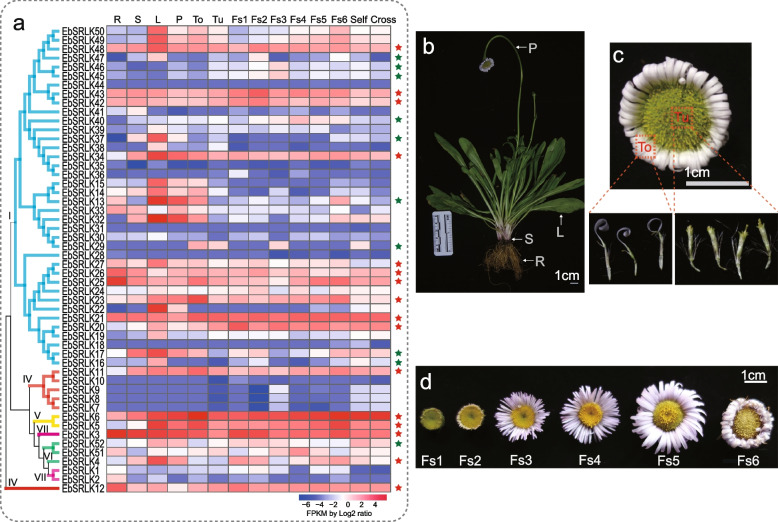
Expression profiles of *SRLKs* in different tissues and at different flowering stages of *Erigeron breviscapus*. **a** The expression patterns of 52 *EbSRLKs* in different tissues and at different flowering stages. R, root; S, stem; L, leaf; P, peduncle; To, tongue flower; Tu, tubular flower; Fs, flower stage; Self, self-pollinated; Cross, cross-pollinated. The expression levels of genes are presented using FPKM fold-change values transformed to Log2 format. The data of 52 *EbSRLKs* were extracted from our RNA-Seq data, The *SRLKs* with high expression levels are highlighted with red stars. The *SRLKs* with higher expression level in the self-pollinated sample than in the cross-pollinated sample are highlighted with green stars. (b-d) The phenotypes of multiple organs and flower developmental stages that samples were collected. **b** Plant organs of *Erigeron breviscapus*. **c** Tongue flowers and tubular flowers. **d** Six flowering stages. Bar = 1 cm in each panel

To explore the spatiotemporal expression patterns of each *EbSRLK* gene, the FPKM values of the identified *EbSRLK* genes were extracted from our previously reported *E. breviscapus* Expression Database [47]. The expression levels (FPKM values) in different tissues and at different flowering stages were presented as heat maps (Fig. 4a). Based on this analysis, some genes, such as *EbSRLK44*, *EbSRLK35*, *EbSRLK31*, *EbSRLK28* and *EbSRLK18*, showed low expression levels in all tissues examined. In contrast, other genes, such as *EbSRLK48*, *EbSRLK43*, *EbSRLK21*, *EbSRLK6* and *EbSRLK3*, were constitutively expressed in every tissue or every flowering stage investigated (Fig. 4a). Compared with other flowering stages, *EbSRLK43* and *EbSRLK48* had high expression levels at Fs2; *EbSRLK29*, *EbSRLK45* and *EbSRLK46* had high expression levels at Fs3; and *EbSRLK40* and *EbSRLK51* had high expression levels at Fs4 (Fig. 4a, d).

As a typical Asteraceae family species, *E. breviscapus* plants have dense rosette leaves, tubular flowers and tongue flowers in the floral organs (Fig. 4b, 4c). Since *SRK* genes are considered to be key genes that control the recognition and initiation of the self-incompatibility response in typical SSI species (such as the Brassicaceae family), in this study, we expected to identify *EbSRLK* genes that show high expression levels in *E. breviscapus* floral organs relative to other vegetative organs. However, we did not find a single *EbSRLK* gene with a significantly elevated expression level in floral organs based on the expression profile of 52 *EbSRLKs* (Fig. 4a), except *EbSRLK23* and *EbSRLK6*, which showed a slightly higher expression level in tongue flowers. By comparing the transcriptomic data of self-pollination and cross-pollination samples, a total of 10 *EbSRLKs* genes (*EbSRLK13, 16, 17, 29, 37, 40, 45, 46, 47,* and *52*) were identified with higher expression levels in self-pollination than in cross-pollination samples. Interestingly, most of these 10 *EbSRLK* genes had low expression levels in flowers at different stages (Fig. 4a).

To further confirm the expression patterns of *EbSRLK* genes from transcriptomic profiling data, 16 *EbSRLK* genes with high expression levels from the transcriptional profiles in different tissues were selected and analyzed using qRT-PCR, with *β-ACTIN* as the reference gene (Supplementary Figure S4, Supplementary Table S5). Totally, 9 *EbSRLK* genes were successfully amplified. *EbSRLK3*, *EbSRLK12* and *EbSRLK26* showed high expression in roots. *EbSRLK34* and *EbSRLK48* showed high expression in leaves and relatively low expression in floral organs. *EbSRLK6* and *EbSRLK23* showed high expression levels in tongue flowers and showed increased expression level as flowers developed. At 6 flowering stages, both *EbSRLK26* and *EbSRLK43* showed a peak expression level at Fs2 (Supplementary Figure S4). In general, the qRT-PCR results confirmed the expression profiles of *EbSRLK* genes from the transcriptomic data.

### Subcellular location of *EbSRLK* genes

**Fig. 5:**
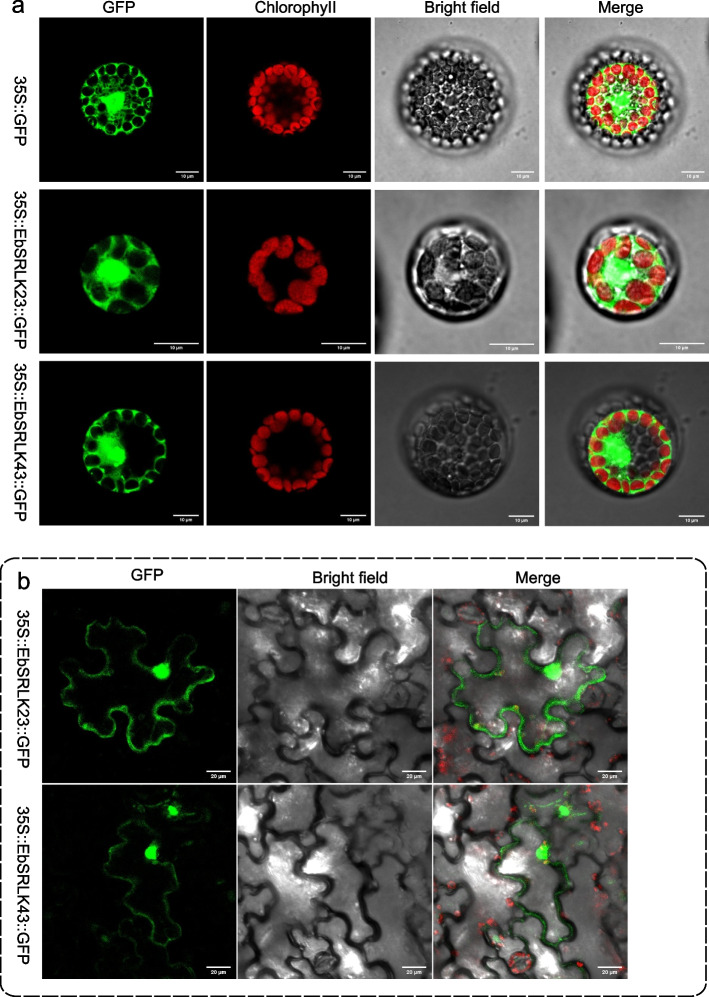
Subcellular localization of EbSRLK23 and EbSRLK43. **a** Subcellular localization in *Arabidopsis thaliana* protoplasts. The recombinant vector *35S::EbSRLK23:GFP* and *35S::EbSRLK43:GFP* and the vector control *35S::GFP* was transfected into Arabidopsis protoplast cells individually. The fluorescence was observed under a laser scanning confocal microscopy. Bar = 10 μm. **b** Subcellular localization of EbSRLK23 and EbSRLK43 proteins in tobacco leaf epidermal cells. Fluorescence images were obtained from confocal microscopy. Merge means image GFP merged with its bright-field photograph in the same cell. Bar = 20 μm

To further confirm the predicted subcellular localization of EbSRLK proteins, the coding sequence (without stop codon) of two *EbSRLK* genes, *EbSRLK23* and *EbSRLK43*, which showed high expression levels in tongue flowers and Fs2 flowers, respectively, were amplified and ligated to fuse with the reporter gene *eGFP* under the control of the CaMV35S promoter. Subcellular localization in transfected *Arabidopsis thaliana* (L.) Heynh protoplasts showed that both EbSRLK23 and EbSRLK43 were detected on the plasma membrane (Fig. 5a). To confirm the results in protoplasts, transient expression of the two constructs was carried out in epidermal cells of tobacco leaves. Results showed that strong fluorescent signals for both EbSRLK23 and EbSRLK43 were detected on the plasma membrane (Fig. 5b).

## Discussion

Previous studies demonstrate that SRKs are crucial in SSI. SRK cloning and functional characterization have been reported in numerous Brassicaceae plants. Furthermore, identification of SRK gene families has been reported in *A.thaliana*, *Brassica napa* and *B*.*oleracea*. Though SI extensively exits in Asteraceae, except sporadic reports on *SRK* gene cloning, no systematic characterization of *SRK* gene families has been reported in Asteraceae. In a previous transcriptomic comparative study of *E. breviscapus*, we identified 230 differentially expressed genes potentially involved in SI. RNA-Seq analysis revealed that some genes (such as *SRLK*, *MLPK* and *KAPP*) are upregulated in self-pollinated flowers but not in cross-pollinated flowers. In contrast, other genes (such as *THL* and *CaM*) exhibit an opposite pattern. Still some genes (such as *Exo70A1*) have little or no significant changes in different pollinated flowers [48]. We further cloned *SRLK1* gene using RACE and analyzed the expression pattern of *SRLK1* [49]. Based on the high quality genome assembly of *E. breviscapus*, we systematically analyzed and characterized the *SRLK* gene family in this study.

### *SRLK* gene family in *E. breviscapus*

Self-incompatibility prevents inbreeding depression caused by self-pollination, thus is very important in the inheritance and evolution of flowering plants. Understanding the mechanism of self-incompatibility is of great significance in plant breeding and crop production [50]. In most SSI plants, *SRK* is specifically expressed on the stigma and is the only important female determinant [51, 52]. Previous studies have identified the *SRK* genes in *B. oleracea* [53], *A. thaliana* (Cruciferae) [54], *Capsella grandiflora* [55], *P. spp* (Rosaceae) [56], *Ipomoea trifida* (Convolvulaceae) [57], *S.squalidus* (Asteraceae) [24–29] and *Corylus heophylla* × *Corylus avellana* (Betulaceae) [58]. In this study, we systematically identified the *SRLK* genes in the Asteraceae plant *E. breviscapus*. Fifty-two *EbSRLK* genes were identified in the *E. breviscapus* genome. This is the first systematic analysis and comparison of the *SRLK* gene family in *E. breviscapus* and in Asteraceae.

### Expression data analysis shows possible involvement of *EbSRLKs *in *E. breviscapus* SI response

Our analysis showed marked polymorphism in the *EbSRLK* genes (Fig. 3). The polymorphism may be due to the strong negative frequency-dependent selection occurred at the *S*-locus [59, 60]. In many plants, the *S*-locus alleles are highly polymorphic [61–64], such as in *A. halleri* and *A. lyrate* [65], and *B. neustriaca* [66]*.* In *Brassica* genus, the sequences of the SRK gene family are complex [67]. The diversity of *SRK* alleles in Brassica plants is as high as 35% [68]. *SRK* genes can maintain at such a high diversity level in the population for a long period of time, which could be beneficial for finely regulated self-incompatibility.

SRKs are membrane proteins, containing three distinctive domains: an extracellular domain (S-domain), a transmembrane domain and an intracellular domain with kinase activity [69]. This allows the SRK proteins to play important roles in the extracellular recognition events between pollen and pistil and the intracellular signal transduction pathway that initiates self-incompatibility [70, 71]. Most EbSRLKs contain at least one transmembrane domain (Supplementary Table S2). This means that most EbSRLKs can be positioned on the cell membrane to exert their functions. The SRK proteins in Brassica contain five subdomains. Among them, S_locus_glycoprotein (SLG, PF00954) is a necessary domain for SSI [62]. The 52 *EbSRLK* genes contain part or all of the five structural or functional domains. For example, *EbSRLK2* contains all five structural domains, and *EbSRLK11* contains only three structural domains, but the SLG domain is a structural domain in all EbSRLKs*.*

Phylogenetic analyses of the SRLK sequences in *E. breviscapus* and two other self-incompatibility species indicate that like in *B. oleracea* and *Pyrus communis*, majority of SRLKs in *E. breviscapus* form an independent group. However, there are also 14 EbSRLKs that are scattered in different clades, predominantly clustered with PsSRLKs or BoSRLKs*.* Both *A. thaliana* and *B. oleracea* are SSI species in the Cruciferae [72], and *P. communis* is a GSI species in the Rosaceae [73]. Therefore, although some researchers used dyeing methods to observe the phenotypes and believed that *E. breviscapus* is an SSI species [74], we still need further solid evidence to determine whether it is indeed an SSI species.

Tissue- or organ-specific expression patterns usually reflect the corresponding biological functions of genes. In *B. oleracea,* reverse transcription polymerase chain reaction (RT-PCR) was used to detect the expression of *SRK* family members in stigma, leaf and root tissues and the *SRK* expression was found to be stigma-specific [75]. In our experiments, except that the expression levels of *EbSRLK5* and *EbSRLK*9 are too low to be detected, other *EbSRLKs* are expressed in roots, stems, leaves and flowers, indicating that *EbSRLKs* may have other functions besides involving in self-incompatibility. In *Corylus heterophylla* × *Corylus avellana*, the *ChaSRK* gene was also found to be expressed in non-flower tissues, with the highest expression level in root tips [49]. Twenty-four hours after pollination, 10 *EbSRLK*s genes exhibit higher expression levels in self-pollinated flowers than in cross-pollinated flowers, suggesting these *EbSRLK* genes may play a role in self-incompatibility. Other *EbSRLK*s genes do not exhibit differential expressions between self- and cross-pollinated flowers, suggesting they may play roles in other aspects of growth and development in *E. breviscapus*. Our study provides clues for future research on deciphering the complex functions of *SRK* genes in *E. breviscapus*.

### Evolutionary expansion of the *E. breviscapus SRLK* gene family

Phylogenetic comparison indicates that EbSRLKs account for the majority (38 out of 41) of Clade I, while Clade III only contains SRLKs from *P*. *communis*. The SRLKs from *B. oleracea* constitute 68% of Clade VII members. This phenomenon is possibly due to the differences among the three species. Gene expansion could be caused by whole genome duplication (WGD), tandem and segmental duplication at the chromosomal level, and transposon-mediated duplication. The main mechanism of gene duplication was segmental duplication and tandem duplication, The genes in duplicate pairs may go through positive selection and gene conversion after the duplication [76, 77]. In previous studies, several rounds of whole genome duplication in rice genome were reported and this resulted in many duplicated genes [78, 79]. On a genome scale it was found that the rice and poplar TLP family should have expanded mainly through segmental duplication events, rather than through tandem duplication and replicative transposition events [79, 80]. Asteraceae is estimated to originate at ~ 83 Mya. During the evolution, 41 WGDs have been detected in Asteraceae species, accompanied by morphological changes including herbaceousness and capitulescence with multiple flower-like capitula, often with distinct florets and scaly pappus/receptacular bracts. Multiple WGDs might have contributed to the survival of early Asteraceae species by providing new genetic pools to support morphological adaptation. The resulting competitive advantage for adapting to different niches would have increased biodiversity in Asteraceae, making it one of the largest flowering plant families [49]. Duplication events, especially segment type, could increase the gene family members in plants and mutations in the regulatory regions such as promoter site can modify the expression levels and function of new members [81, 82]. Our results indicate that 92.68% of EbSRLK are clustered in Clade I. This biased distribution leads us to speculate whether one or more gene duplication events occurred during the evolution of *E. breviscapus*. Chromosomal positioning analysis suggests multiple tandem duplications of *EbSRLK* gene clusters. Transposon-mediated duplication might also lead to *EbSRLK* gene expansion as exemplified by *EbSRLK22* and *EbSRLK14*. In addition, chromosomal segmental shift is another factor in gene duplication. For example, *EbSRLK33* and *EbSRLK28* share apparent co-linearity. Similar pattern can be found between the gene cluster of *EbSRLK44/48/45/37* and *EbSRLK40* and *EbSRLK41*.

To understand the exact functions of the *EbSRLK* genes, we will determine the diverse functionality of the *EbSRLK* genes by constructing overexpression constructs of individual *EbSRLK* genes and generate transgenic plants, or using the CRISPR/Cas9 system for *EbSRLK* function validation.

## Conclusions

A total of 52 *EbSRLKs* genes were identifed and their distribution on chromosomes, gene structure, conserved motif, evolutionary relationships and Subcellular location were analyzed. The results show that *EbSRLKs* are highly conserved and contains a conserved *S*-locus glycoprotein domain, which is the special sequences protein structure of the *SRLKs* family. *EbSRLKs* with higher expression level in self-pollinated sample than in cross-pollinated sample. which deserve further attention and research, and these results implyed that *EbSRLKs* genes may involved in the SI reaction of *E.breviscapus*. This study provides useful information for further study of the function of the *EbSRLKs* genes and SI mechanism in Asteraceae.

## Supplementary Information

**Additional file 1.**

## Data Availability

The supporting data such as the high-quality reads produced in this study have been deposited in the NCBI SRA database, an open access repository (accession number: PRJNA 379607, https://www.ncbi.nlm.nih.gov/bioproject/PRJNA806708. & accession number PRJNA277583, https://www.ncbi.nlm.nih.gov/bioproject/PRJNA277583).
