# Supplementary material for: Genome-wide identification and characterization of SRLK gene family reveal their roles in self-incompatibility of Erigeron breviscapus
Source: BMC Genomics. 2023 Jul 17;24:402. doi: 10.1186/s12864-023-09485-0 (PMC10353254; doi:10.1186/s12864-023-09485-0)
Supplement: Supplementary file 1 — Additional file 1. [file 12864_2023_9485_MOESM1_ESM.zip › Additional File/SupFigure 1.pdf]

a

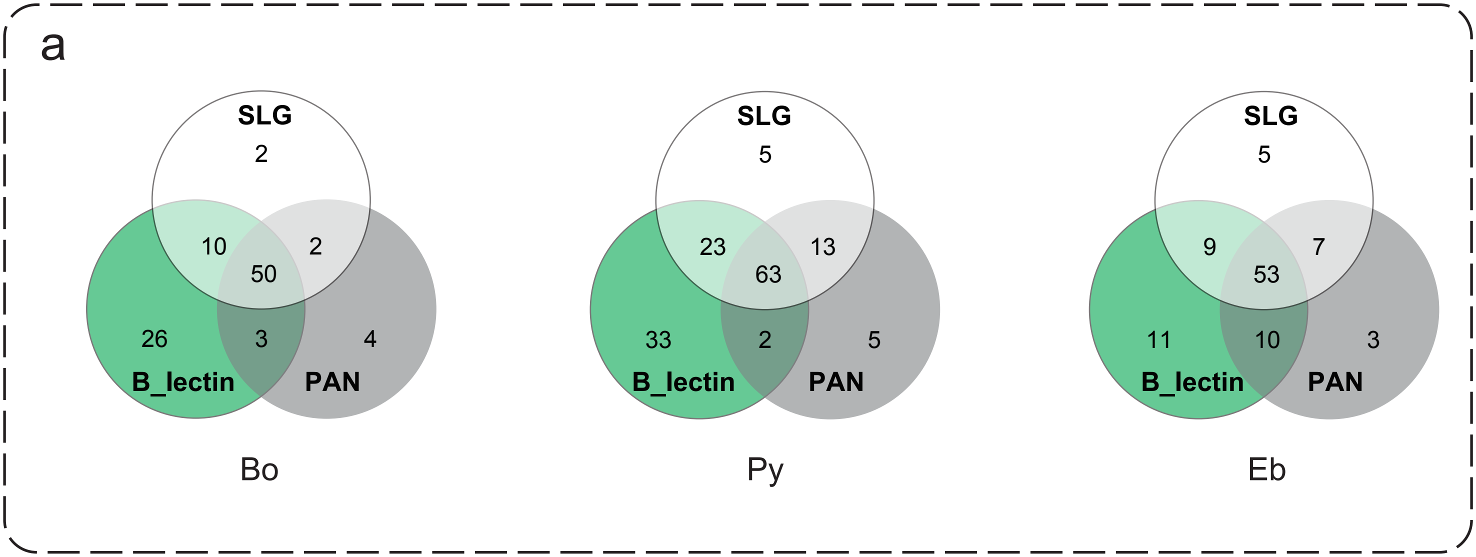
SLG
2
10
2
50
26
3
4
B_lectin
PAN
Bo
SLG
5
23
13
63
33
2
5
B_lectin
PAN
Py
SLG
5
9
7
53
11
10
3
B_lectin
PAN
Eb

b

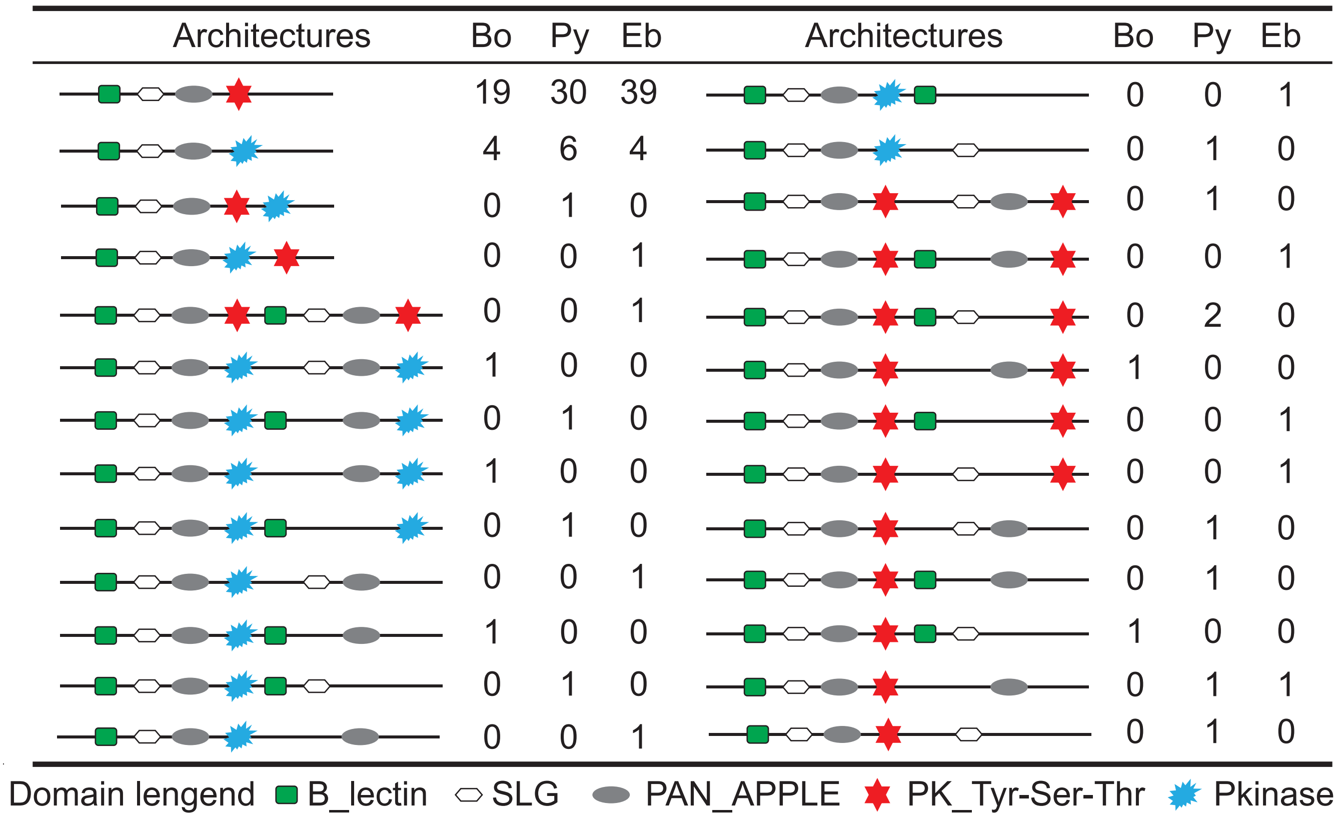
Architectures
Bo
Py
Eb
Architectures
Bo
Py
Eb
19 30 39
4 6 4
0 1 0
0 0 1
0 0 1
1 0 0
0 1 0
1 0 0
0 1 0
0 0 1
1 0 0
0 1 0
0 0 1
0 0 1
0 1 0
0 1 0
0 0 1
0 2 0
1 0 0
0 0 1
0 0 1
0 1 0
0 1 0
1 0 0
0 1 1
0 1 0
Domain lengend
B_lectin
SLG
PAN_APPLE
PK_Tyr-Ser-Thr
Pkinase
