# Supplementary material for: Genome-wide identification and characterization of SRLK gene family reveal their roles in self-incompatibility of Erigeron breviscapus
Source: BMC Genomics. 2023 Jul 17;24:402. doi: 10.1186/s12864-023-09485-0 (PMC10353254; doi:10.1186/s12864-023-09485-0)
Supplement: Supplementary file 1 — Additional file 1. [file 12864_2023_9485_MOESM1_ESM.zip › Additional File/SupTable legend.docx]

SupTable1. Identified Genes from three species

List of identified genes which contain B_lectin, SLG and PAN domains from three species.

SupTable2. Characterization of the 52 *EbSRLK* genes identified in *Erigeron breviscapus* genome

SupTable3. Synteny Block identified in *Erigeron breviscapus* genome

SupTable4 The *Ka/Ks* ratios for tandemly duplicated *EbSRLK* genes

SupTable5. Primer information used in qRT-PCR
