# Supplementary material for: Genome-wide identification and characterization of SRLK gene family reveal their roles in self-incompatibility of Erigeron breviscapus
Source: BMC Genomics. 2023 Jul 17;24:402. doi: 10.1186/s12864-023-09485-0 (PMC10353254; doi:10.1186/s12864-023-09485-0)
Supplement: Supplementary file 1 — Additional file 1. [file 12864_2023_9485_MOESM1_ESM.zip › Additional File/Supplementary figure legend.docx]

**Figure S1. Identification and domain architectures of *SRLKs* in three species.**

(a) Identification of *SRLKs* in *Brassica oleracea* (Bo), *Pyrus communis* (Py) and *Erigeron breviscapus* (Eb). The hidden Markov model (HMM) profiles PF01453 (B_lectin), PF08276 (PAN) and SLG (PF00954) were extracted from Pfam database and used to search the local protein databases of three species.

(b) Number and domain architectures of SRLKs from the three species. B_lectin, SLG and PAN domains are represented by green cube, white hexagon and gray oval, respectively. Two types of kinase are represented by different symbols.

**Figure S2. Prediction of transmembrane domains (TM) in 52 EbSRLK proteins.**

The value on the ordinate axis is the probability value of each amino acid on the horizontal axis that is localized inside the membrane, outside the membrane, and TM helix. The red rectangles represent the transmembrane spiral structure.

**Figure S3. Motif compositions and distribution of conserved motifs in 52 EbSRLK proteins.**

The conserved motifs in 52 EbSRLK proteins were identified by MEME. Gray lines represent non-conserved sequences and each motif is indicated by numbered boxes and by different colors. Motifs that could be scanned but could not be confirmed from MEME sites are labeled with grey inverted triangles. Seqlogo plots of 8 identified motifs were generated using batch MEME motifs viz program. The lengths of the motifs in each protein are proportional.

**Figure S4. Quantitative RT-PCR analysis of expression levels of nine *EbSRLK* genes in different** ***Erigeron breviscapus* tissues.**

Total RNAs were isolated from different *Erigeron breviscapus* tissues. Relative expression levels of *EbSRLK* genes in different *Erigeron breviscapus* tissues are shown as the percentage of *Erigeron breviscapus* *ACTIN* expression level. Data presented in the quantitative RT-PCR analysis are mean values with standard deviation of three biological replicates of plant materials and three technical replicates in each biological sample. R, root; S, stem; L, leaf; P, peduncle; To, tongue flower; Tu, tubular flower; Fs, flowers at different developmental stages as shown in Figure 4.
