# Supplementary figures and images for: Genome-wide identification and characterization of SRLK gene family reveal their roles in self-incompatibility of Erigeron breviscapus
Source: BMC Genomics. 2023 Jul 17;24:402. doi: 10.1186/s12864-023-09485-0 (PMC10353254; doi:10.1186/s12864-023-09485-0)

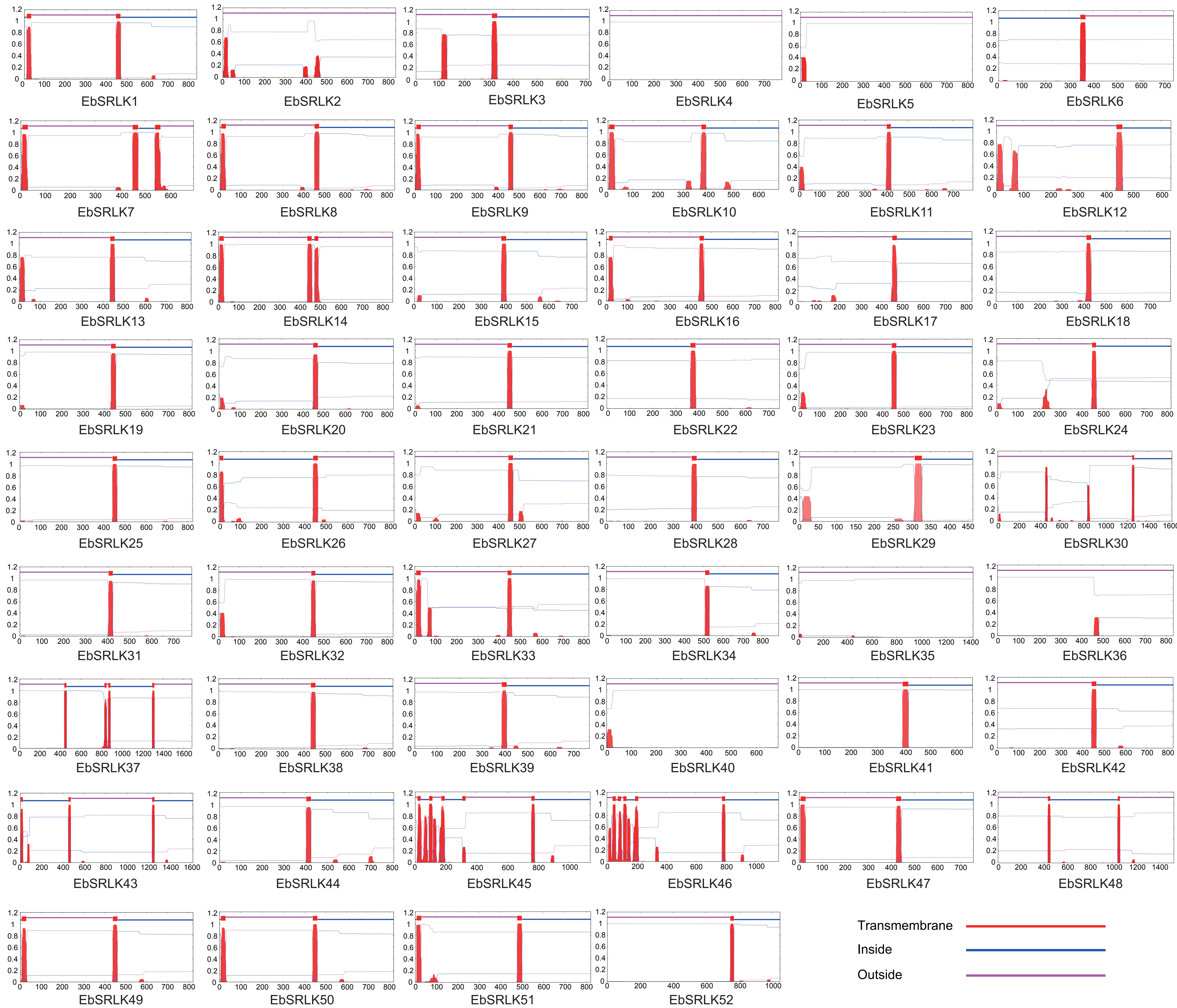

Supplement: Supplementary file 1 — Additional file 1. [file 12864_2023_9485_MOESM1_ESM.zip › Additional File/SupFigure 2.pdf]

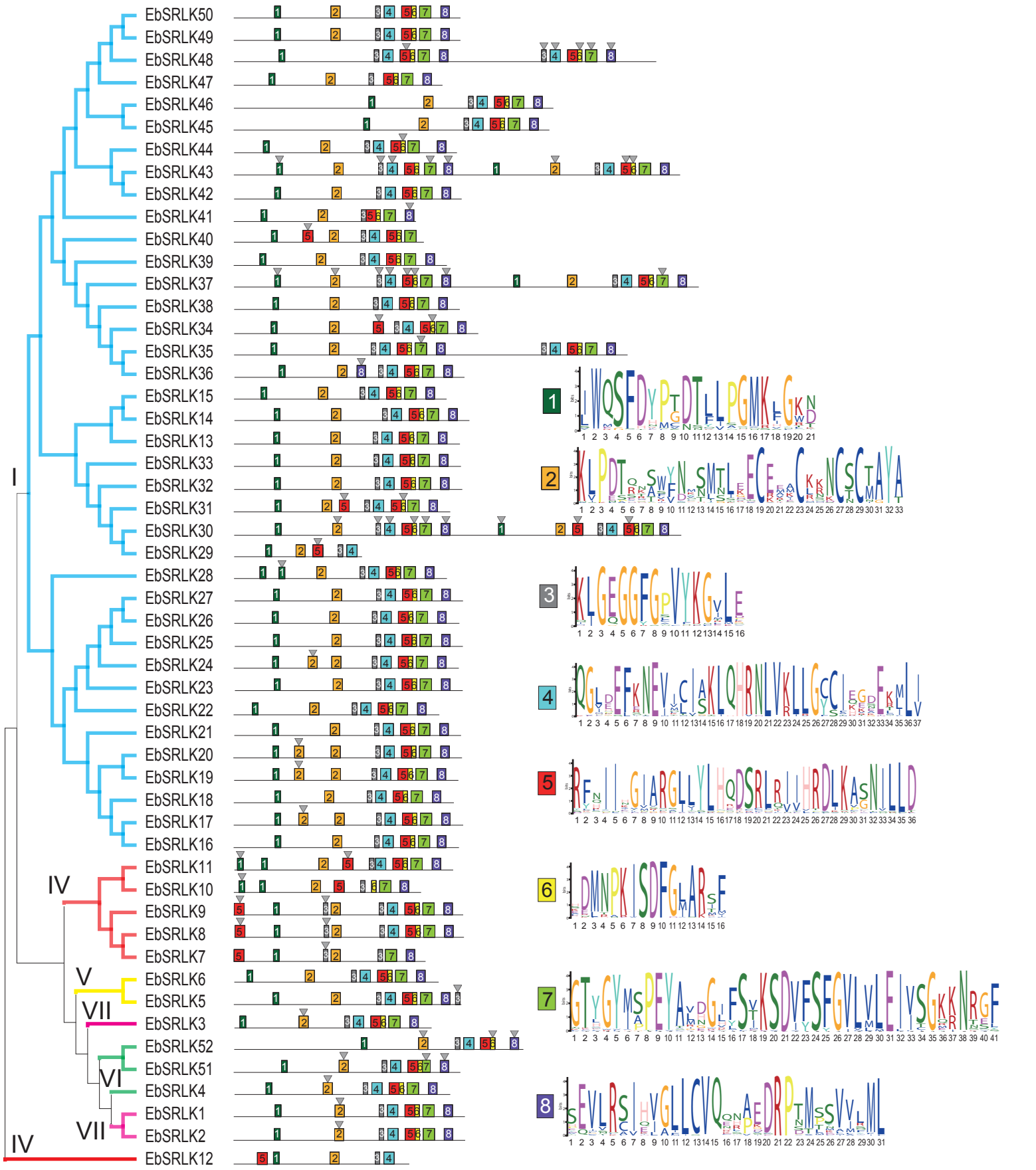

Supplement: Supplementary file 1 — Additional file 1. [file 12864_2023_9485_MOESM1_ESM.zip › Additional File/SupFigure 3.pdf]

*EbSRLK3*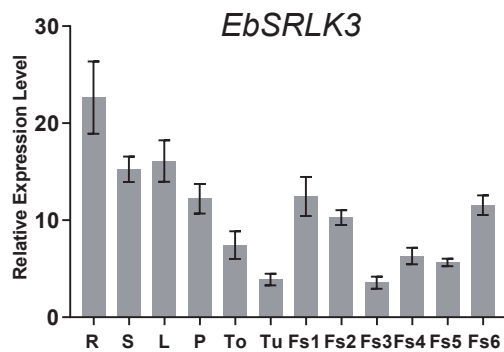*EbSRLK6*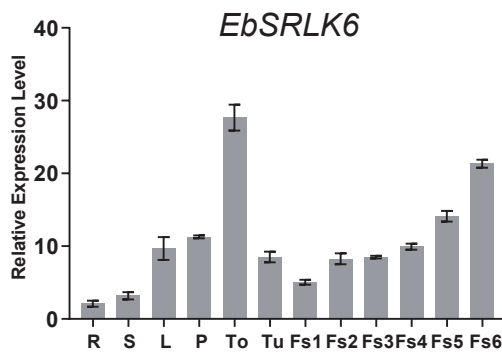*EbSRLK12*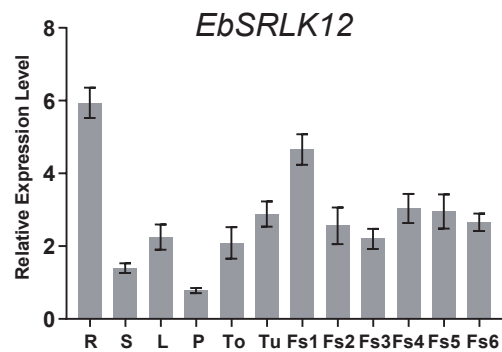*EbSRLK21*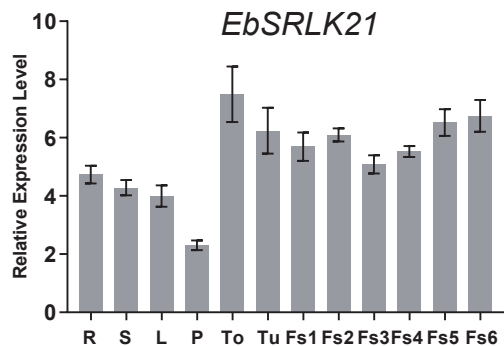*EbSRLK23*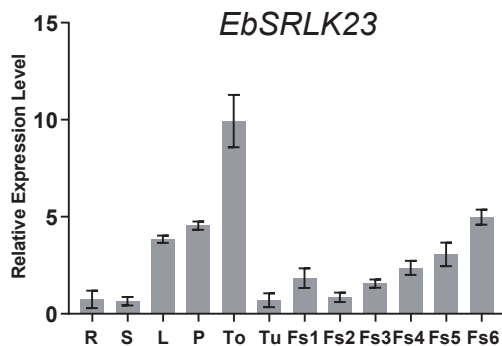*EbSRLK26*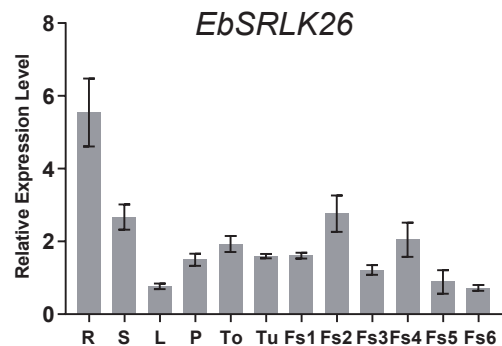*EbSRLK34*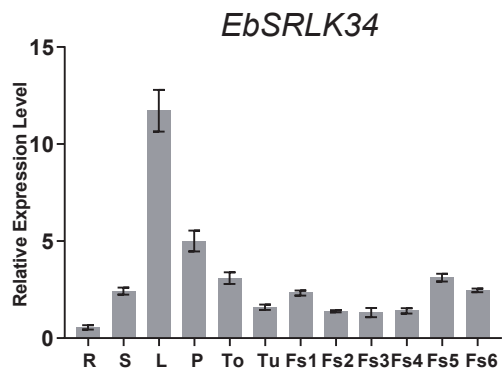*EbSRLK43*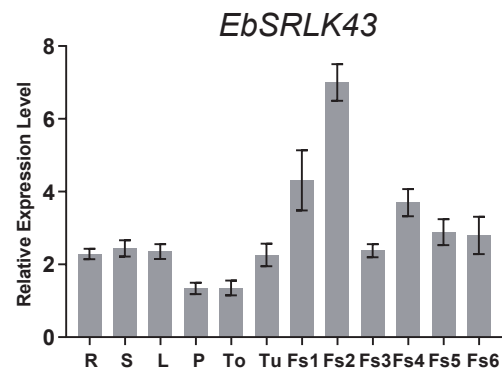*EbSRLK48*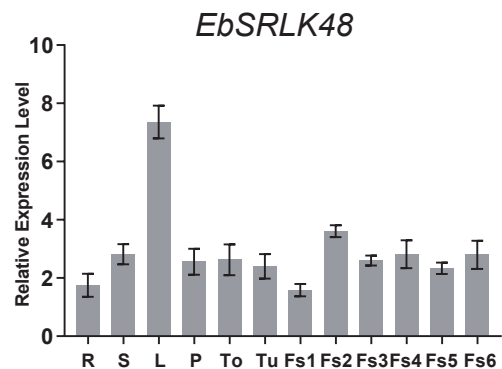

Supplement: Supplementary file 1 — Additional file 1. [file 12864_2023_9485_MOESM1_ESM.zip › Additional File/SupFigure 4.pdf]
